# Editorial: Innovations in natural dye production: bridging tradition and modern technology

**DOI:** 10.3389/fpls.2025.1685273

**Published:** 2025-09-02

**Authors:** Janne Rojas

**Affiliations:** “Organic Biomolecules” Research Group, Faculty of Pharmacy and Bioanalysis, University of Los Andes, Mérida, Venezuela

**Keywords:** natural dyes, innovation, plant research, extraction methods, biologic activity

Natural dyes have been used for millennia in the cultural practices of communities worldwide. In this sense, plants are the most important sources of natural dyes. Around 500 plant species have been identified as potential sources of dye and these are produced from the root, stem, bark, leaves, flowers, fruits, seeds, or wood and exist in a variety of colors, such as red, yellow, blue, black, brown, and their combinations (Miranda et al., 2025). It is well known that plant pigments are chemical compounds biosynthesized by plants. Anthocyanins, carotenoids, chlorophylls, among others, are examples of natural dyes. However, these natural pigments are also beneficial to health since possess antioxidant, anti-inflammatory, antibacterial, anticancer, and even antiallergic properties. These attributes make them promising compounds for the prevention of chronic diseases and the promotion of well-being (Yadav et al.).

In this regard, anthocyanins are pigments responsible for the coloration of fruits, vegetables and petals in many flowers, and phenolic compounds are recognized not only as pigments but as natural antioxidants as well. Their presence is mainly related to the color of flowers, however, are being studied as possible sources for producing food additives because of their bioactive properties (Ohifueme and Olatunde, 2024).

Nowadays, natural dyes are considered as a sustainable alternative to synthetic dyes due to their eco-friendliness and potential health benefits since synthetic dyes, widely used for their stability and low cost, have been associated to health risks, including allergic reactions, toxic effects, and even long-term carcinogenicity. In recent years, the development of green extraction and stabilization technologies has played a crucial role in overcoming natural dyes extraction limitations. Methods such as ultrasound assisted extraction (UAE), microwave-assisted extraction (MAE), and supercritical fluid extraction (SFE) are known for their efficiency, sustainability, and ability to preserve the bioactive properties of pigments (Miranda et al., 2025).

Despite the growing interest in natural dyes, challenges related to their stability, seasonality, and extraction efficiency continue to limit their commercial use. One of the contributions featured in this volume is the isolation of a food grade antioxidant red dye from royal poinciana flower. The aim this study was to evaluate the extraction process by magnetic stirrer assisted techniques of pigments from royal poinciana flower with different shades of red, and to determine the content of hydrolysable polyphenols, condensed tannins, and antioxidant potential by DPPH, ABTS and FRAP tests (Isolation of food grade antioxidant red dye from Royal poinciana flower petals for functional food, 2025).

Another interesting research described in this chapter versed on the sustainable dyeing of cotton, silk, and leather using the *Bixa orellana* seeds, commonly known as annatto. The processes of extraction, optimization, and the subsequent assessment of antibacterial activity highlight not only the vibrant colors achievable through these natural sources but also the potential antibacterial properties that can enhance the functionality of textiles in health-conscious markets (Sustainable dyeing of cotton, silk and leather using natural dye from Bixa orellana seeds: Extraction, optimization and assessment of antibacterial activity, 2025).

The efficacy of ultrasound-assisted extraction of *B. vulgaris* and its potential to reduce bacterial load has also been described. The results of this study indicated that the dyed material exhibited antibacterial properties against the skin pathogens such as, *Staphylococcus* sp, *Vibrio* sp., *Pseudomonas* sp., *Klebsiella* sp., and *Micrococcus* sp (Eco-friendly natural dye extraction technique using Beta vulgaris and its active dyeing on cotton, Silk, leather and its antibacterial ability assessment, 2025). Further innovating the field, the ultrasonic extraction of antibacterial dye from *Basella alba* fruit is highlighted as a sustainable approach to dyeing. This technique eliminates the need for mordants, traditionally used to fix dyes, thereby reducing environmental impact while still achieving durable and vibrant colors on cotton and silk fabrics, as well as leather products. In this study, the extracted dye *Basella alba* fruit exhibited significant antibacterial activity against *Pseudomonas* sp. with a MIC value of 1.56 mg/mL (Ultrasonic extraction of antibacterial basella alba fruit dye for dyeing cotton, silk fabric, and leather without mordant: A sustainable approach, 2025).

The studies described in present chapter have demonstrated that plants have great potential to be applied as natural food dyes, offering an excellent alternative to synthetic dyes, however, more investigations need to be carried out on this subject.
